# Modular tumor endoprostheses in surgical palliation of long-bone metastases: a reduction in tumor burden and a durable reconstruction

**DOI:** 10.1186/1477-7819-12-330

**Published:** 2014-11-07

**Authors:** Marcel-Philipp Henrichs, Juliane Krebs, Georg Gosheger, Arne Streitbuerger, Markus Nottrott, Tim Sauer, Steffen Hoell, Gurpal Singh, Jendrik Hardes

**Affiliations:** Department of Orthopaedic Surgery, University Hospital Muenster, Albert-Schweitzer-Campus 1, Building A1, 48149 Muenster, Germany; University Orthopaedics, Hand and Reconstructive Microsurgery Cluster, National University Health System, Singapore, Singapore; Department of Oncology, University Hospital Muenster, Albert-Schweitzer-Campus 1, Building A1, 48149 Muenster, Germany; Department of Orthopaedic Surgery, Paracelsus Hospital Osnabrueck, Am Natruper Holz 69, 49076 Osnabrueck, Germany

**Keywords:** Bone metastasis, Modular, Surgical reconstruction, Survival, Tumor burden, Tumor endoprostheses

## Abstract

**Background:**

Surgical treatment of bone metastases has become increasingly important as patients live longer with metastatic cancer and one of the main aims is a long-lasting reconstruction which survives the patient. Conventional osteosynthesis may not be able to achieve this objective in the context of modern day cancer care.

**Methods:**

This study evaluates the oncological outcomes, treatment-related complications, and function after resection of metastases and reconstruction with modular tumor endoprostheses in 80 patients. All patients who underwent surgical treatment with modular tumor prostheses for bone metastases from 1993 to 2008 were traced by our tumor database and clinical information was recorded from patient case.

**Results:**

Mean age was 63 years. The most common primary tumors were renal cell (47%), breast (21%), and lung (8%). The proximal femur was affected in 45%, proximal humerus in 26%, and the distal femur in 17% of cases. In 22 cases, the tumor prosthesis was implanted during a revision operation. Mean overall survival after surgery was 2.9 years. Overall survival rate was 70% at one year and 20% at five years. Implant survival was 83% after one year and 74% at five years. Overall rate of operative revision was 18%.

**Conclusions:**

Our data collectively suggest that despite higher costs, implantation of modular tumor endoprostheses may be a suitable treatment for bone metastases with a low complication rate and rapid improvement in function in appropriately selected patients.

## Background

Surgical treatment of bone metastases has become increasingly important in view of the longer life expectancy of patients with metastatic cancer, coupled with advances in musculoskeletal oncology [1, 2]. Estimation of prognosis and survival is subjective. It has been shown that estimation of survival in cancer patients may be correct only in 18% and underestimated in 43% of patients [3]. In addition, current decision-making in metastatic cancer patients needs to consider not only prognosis and survivorship, but also quality of life and function [1, 4]. The skeleton is the third most common site of metastases, and metastases are the most common tumors of bone. At least every third to fifth patient with cancer develops bone metastases [1]. One of the most common complications of long bone skeletal metastases is a pathological fracture and even though long bones of the extremities are not the most common site of metastases, long bone metastases often lead to loss of function, pain, and loss of ambulation for the patient [2].

Options for surgical management include osteosynthesis (fixation with an intramedullary nail or plate, in combination with adjuvants such as bone cement), endoprostheses (joint replacement prostheses), or tumor endoprostheses (modular or conventional non-modular components). Recent advances in musculoskeletal oncology question the validity of osteosynthesis for surgical palliation of the cancer patient with bone metastases in today’s context of multimodal cancer care and increased survival. The line between palliative and curative intent has also shifted, and the there is growing evidence that local control of metastases might have a positive impact on prognosis [5]. Surgical palliation in metastatic bone disease has conventionally been osteosynthesis with a rigid construct such as an intramedullar nail. Ideally, the construct should outlive the patient, surgery should provide effective and fast pain relief and ambulation, and there should be an improvement in survival after surgery, particularly for solitary metastases [6]. With increased survival of patients with long bone metastases, tumor recurrence, failure of the fixation, and continuing pain are increasing in incidence and thus the idea of the osteosynthetic construct surviving the patient may not be able to keep up with rapid advances in multimodal treatments which allow patients with bone metastases better survival. There has to be a frameshift in thinking with regards to optimal surgical management of the patient with metastases, with a greater emphasis on tumor resection (particularly for solitary metastases) and reconstruction with longer-lasting options [7, 8]. We hypothesize that compared to conventional osteosynthetic devices, modular tumor endoprosthetic reconstruction provides early mobilization, fast relief of pain, and a longer implant survival in the context of current multimodal and multidisciplinary treatment despite higher costs, and may be a more suitable procedure in appropriately selected patients.

## Methods

Patient data was retrieved from a prospectively maintained tumor database from 1993 to 2008. There were 80 patients (82 surgeries) who underwent long bone metastatic tumor resection and reconstruction with a modular tumor MUTARS endoprosthesis (Implantcast GmbH, Buxtehude, Germany). There were 30 female and 50 male patients, with a mean age of 63 years ± SD 11.3 (range 34.3 to 83.6). Mean duration of follow-up was 19 months, ranging from 0.5 to 106.8 months. Two patients were lost to follow-up. Metastasis in this cohort occurred from 13 different primary tumors, the most common primaries being renal cell, breast, and lung (Table 1). In addition, in 4 cases, the primary tumor remained unknown. Mean duration from diagnosis to operation was 56 months (range 0.2 to 280.9). There were multiple metastases in 57 patients. Bone and visceral metastases occurred in 39 patients (48.8%), multiple bone metastases in 18 patients (22.5%), and solitary bone metastases in 23 patients (28.8%). Early metastases (<2 years after index diagnosis) occurred in 49 of 82 cases (59.8%). Late onset metastases (2 to 14 years after index diagnosis) occurred in 33 of 82 cases (40.2%). Overall, pathological fractures occurred in 46 patients (56.1%). The most common site of metastasis was the proximal femur, followed by the proximal humerus (Table 2). Mean duration between first operation and implantation of the MUTARS tumor endoprosthesis in these patients was 28 months (range 2 months to 13 years). In the majority of cases we used silver coated implants (Table 2).

**Table 1:** **Metastases**

Primary tumors	
Renal	46.3%
Breast	21.3%
Lung	7.5%
Prostate	5.0%
Cancer of Unknown Primary (CUP)	5.0%
Malignant melanoma	2.5%
Esophagus	2.5%
Others	10.0%

**Table 2:** **Therapy**

Operative treatment			Prostheses	n =82	100%
**Type of resection**	**n =77**	**100.0%**	Proximal humerus	21	25.6%
Wide	41	53.2%	Distal humerus	1	1.2%
Marginal	14	18.2%	Total humerus	2	2.4%
Intralesional	22	28.6%	Diaphyseal humerus	2	2.4%
			Proximal femur	37	45.1%
**Adjuvant/neoadjuvant treatment**			Distal femur	14	17.1%
**Local radiotherapy**	**n =48**	**58.5%**	Total femur	1	1.2%
Adjuvant	26		Proximal tibia	2	2.4%
Neoadjuvant	13		Diaphyseal femur	1	1.2%
Both	9		Diaphyseal tibia	1	1.2%
**Chemotherapy**	**n =29**	**35.4%**	**Silver coated prostheses**	**51**	**62.2%**
**Preoperative embolization**	**n =13**	**15.9%**	**Cemented implantation**	**n =72**	**87.8%**

The research has been performed in accordance with the declaration of Helsinki. As this retrospective analysis consists of anonymised clinical routine data, the Research Ethics Committee deems the application for and issue of an Ethics approval not necessary (Ethics Committee of the Chamber of Physicians Westfalen-Lippe and the Medical Faculty of the Westfalian Wilhelms University Muenster, reference no. 2014-583-f-N).

Statistical analysis was performed using IBM SPSS statistics version 19.0 (IBM Corporation, Armonk, NY, USA). Overall survival, event-free survival, and prosthetic survival was evaluated by Kaplan-Meier survival analysis (95% confidence interval). Significance was proved with Kaplan-Meier with log rank tests (level of significance <0.05). Estimation of risk was performed using hazard ratios.

## Results

**Figure 1:**
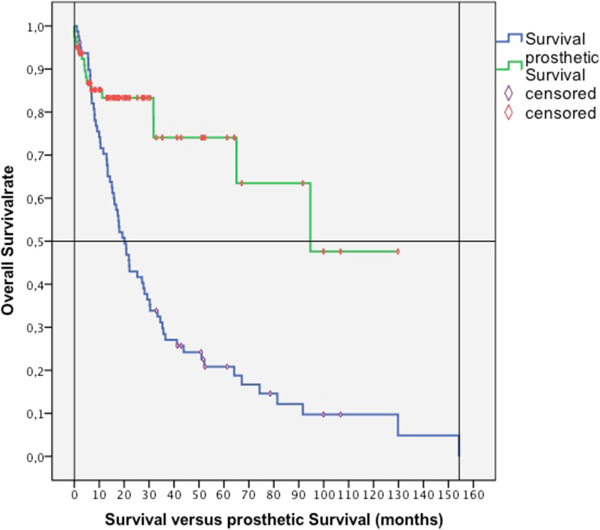
**Survival vs. prosthetic survival (months).**

No patient died within the first month after operation. Overall survival rates were 70% at 1 year post-surgery, 43% at 2 years, 28% at 3 years, and 20% at 5 years (Figure 1). Mean overall survival was 35.2 ± SD 4.6 months (95% CI, 26.2 to 44.2; range, 1.1 to 154.3 months).

The mean survival rates between tumor types, age, and gender are shown in the following table. Age and gender showed no significant influence on overall survival. The differences in overall survival rates between primary tumors were not statistically significant.

With regards to metastasis site, type of primary, extent of metastasis, and time between index cancer and detection of metastasis, the survival rates are presented in Table 3.

**Table 3:** **Survival**

	Mean	1 year survival rate	5 year survival rate
All patients	35 months	70%	20%
Renal cell	38 months	72%	25%
Breast	44 months	87%	27%
Lung	22 months	67%	0%
Solitary bone late onset	49 months	91%	29%
All others	33 months	67%	18%
Revision surgery	53 months		
No revision surgery	30 months		

Nine patients (11.0%) had a local recurrence after operation; 2 of these 9 patients required a second operation. Among the local recurrences, 8 patients had renal cell carcinoma and 1 patient had endometrial carcinoma. The overall recurrence rate of all renal cell carcinomas in our series was 21.6%. After wide resection, the recurrence rate was 13.6% and after marginal or intralesional resection it was 33.3%. Survival of patients with local recurrence: mean: 22.2 months vs. 36.9 months for patients without local recurrence (*P* =0.207).

We defined local progression-free survival as the survival with the endpoints of local recurrence. Progression free-survival after operation for the entire cohort was: mean 34 months ± SD 4.6 (95% CI, 25.0 to 43.2; range, 0.6 to 154.3) and median 19.4 ± SD 2.9 months (95% CI, 13.6 to 25.1). Mean event-free survival in solitary metastasis with wide resection was 41.0 months ± SD 9.6 (95% CI, 22.2 to 59.8; range, 2.7 to 129.8) and after intralesional resection it was 29.2 ± SD 8.1 months (95% CI, 13.3 to 45.1; range, 2.0 to 51.6) Poorer event-free survival was observed in patients with chemotherapy or radiotherapy. For patients without chemotherapy, mean survival was 38.4 ± SD 7.1 (95% CI, 24.4 to 52.4 months; range, 0.6 to 154.3) versus 29.0 ± SD 5.3 months for patients with pre- or/and post-operative therapy (95% CI, 18.6 to 39.3 months; range, 1.1 to 99.9). Patients without adjuvant radiotherapy had an event-free survival of 36.6 ± 6.5 months (95% CI, 23.8 to 49.3 months; range, 0.6 to 154.3) compared to 28.5 ± SD 5.1 months for patients with radiotherapy (95% CI, 18.4 to 38.6 months; range, 1.1 to 106.7).

The overall complication rate in our series was 30.5%. Out of these, wound healing disturbances comprised 7 patients (8.5%) (chemotherapy and radiotherapy having no significant influence). Operative wound revision was necessary in 4 of 7 cases. Loosening of implants (asymptomatic and symptomatic) was observed in 6 patients (7.3%). Operative revision was required in 4 of 6 cases. Periprosthetic infection rate was 7.3% (mean 6 months after operation, proximal femur n =3, distal femur n =2, diaphyseal tibia n =1, infection rate of silver coated prostheses: 3.9% vs. 12.9% in non-silver-coated prostheses, statistically significant, *P* =0.041). Operative revision was required in 4 of 6 cases. Dislocations were seen in 5 patients (6.1%, proximal humerus n =2, proximal femur n =3). Transient palsy of radial nerve was observed in 4 cases. Contracture of the knee joint after implantation of distal femur occurred in 1 patient. One patient had a periprosthetic fracture 2.5 years after implantation of proximal humerus endoprosthesis (Table 4).

**Table 4:** **Overview of postoperative complications**

	Total
**Complications**	25
**Minor**	13
Wound-healing disturbances	7
Transient palsy	4
Joint contracture	1
Periprosthetic fracture	1
**Major**	11
Periprosthetic infection	6
Loosening	6
Sub-dislocation	5
**General complications**	1
ARDS	1
**Revisions**	15

The overall revision rate in our series was 18.3%. Overall median survival in patients who underwent revision surgery was 52.8 ± SD 11.9 months (range, 7.8 to 154.3; 5 year survival rate, 30.0%). Among patients with no revision surgery, overall median survival was 30.4 ± SD 4.6 months (range, 1.1 to 129.8; 5 year survival rate, 17.5%) These differences were statistically significant (*P* =0.036; Table 4).

The revision-free prosthetic survival rate (no operative revision needed) was 83.1% at 1 year, 73.9% at 5 years, and 47.5% at 8 years. Survival of proximal femur endoprostheses was 87.7% at 1 year and at 5 years. Survival of proximal humerus endoprostheses was 94.7% at 1 year and 75.8% at 5 years. The prosthetic reconstruction outlived the patient in 92%. We observed a limb survival rate of 98%.

## Discussion

Studies on tumor prostheses often involve a mixture of primary sarcomas and metastases, or are focused on a particular anatomic region such as the proximal femur or humerus. There is a paucity of outcome studies on the use of modular tumor prostheses in surgical palliation of patients with long bone skeletal metastases with regards to overall survival, event-free survival, and survival of prosthesis, regardless of anatomic region. Our data show a mean post-operative survival of 35 months, which is longer than most reported survival data on surgical palliation of skeletal metastases. The 5-year survival rates we have reported are high compared to currently available data. Our results regarding the survival rates with regards to state of metastases support former reports [3, 9]. Additionally, our data show high survival rates even in multiple metastases (28 months for early-onset multiple metastases, 39 months for late-onset multiple metastases). Thus, modular tumor endoprostheses may be an option in surgical palliation of multiple skeletal metastases as well. In our study, patients with late onset single metastases had the best survival (1 year survival rate 91%, 5 year survival rate 29%). This data is consistent with other studies and thus, in these cases, a curative surgical approach is justified [10, 11].

Most studies show worse survival rates in the context of skeletal metastases with pathological fractures [10, 12, 13]. Our data does not support this phenomenon, and we attribute this to a reduction in tumor burden after surgery. Several authors have reported a higher survival rate after wide resection of metastases [11, 14]. Our data does not show statistically significant differences in survival between wide and marginal resections. However, the overall results of our study support the notion that tumor resection, which is necessary for modular tumor endoprosthesis reconstruction, leads to a reduction in tumor volume and increases survival. In addition, from a reconstruction point of view, implantation of a modular tumor endoprosthesis is a good option in large bony defects, which are often seen after pathological fractures. However, one must understand the limitations of this study, including the nonrandomized and retrospective nature, and therefore the inability to control some potential confounding variables.

There have been conflicting reports on survival among patients with local recurrence of skeletal metastases after surgery. Most studies report lower survival among patients with local recurrence. However, Utzschneider et al. [14] reported a higher survival for patients with local recurrence as patients have to survive a longer time to reach the point of recurrence; they conclude that tumor recurrence might be an indirect indicator for longer survival. The local recurrence rate among skeletal metastases is quoted to be between 3 to 20% [15, 16]. The incidence of local recurrence in our study was 11% and most of the tumors that recurred were renal cell carcinomas. Most series reporting recurrence rates of renal cell carcinomas have similar numbers to those in our study as renal cell carcinoma is not usually radio- or chemosensitive. Our results showed that patients with local recurrence had a mean survival of 22 months, whereas patients without local recurrence had a mean survival of 37 months. However, only 2 patients required revision surgery due to local recurrence. We believe that resection of metastases and reconstruction with modular tumor endoprostheses achieves better oncological and functional outcomes in a selected group of patients as incomplete resection of metastatic lesions is associated with inadequate relief of pain and higher rates of local tumor progression.

Besides oncological outcomes, survival of the modular tumor endoprostheses and the ability of the reconstruction to ‘outlive’ the patient are key outcome indicators of successful surgical palliation in these patients. In our study, 92% of the reconstructions outlived the patient. The proximal femur and proximal humerus were the most common sites of reconstruction and high revision-free survival rates were observed at both locations. In the proximal femur, endoprosthesis survival rate was 88% after one year and after 5 years. In the proximal humerus, endoprosthesis survival rate was 95% after one year and 76% after five years. It is known that patients with bone metastasis have poorer functional outcome compared to primary tumors as most patients with metastases are older and suffer from other diseases. Compared to Gosheger et al. [17], we achieved almost the same scores for reconstructions of the proximal humerus and proximal femur despite the difference of primary vs. secondary tumors. Complication rates in our series were consistent with reported outcomes in the literature. With regards to dislocation of proximal femur endoprostheses, we achieved a rate of 8% compared dislocation rates of up to 20% in the current literature [18]. Reported revision rates of secondary tumors are in the range of 3 to 26% [3, 7, 8, 19, 20]. We achieved a revision rate of 18%, and attribute this to a high rate of reoperation (revision of osteosyntheses), high incidence of pathological fractures, and a high proportion of tumors with longer survival (breast, kidney) in our cohort.

Perhaps the final issue that deserves mention is cost-effectiveness of surgical treatment of bone metastases, and as a subset, the justification of increased cost of treating bone metastases with modular tumor endoprostheses. Skeletal-related events from bone metastatic disease result in significant health resource utilization, imposing a substantial financial burden on health systems. Treatments that delay or prevent skeletal-related events therefore result in considerable cost-savings [21]. Singh et al. [22] have shown that it is more cost-effective to surgically reconstruct metastases around the hip joint in appropriately selected patients, as compared to the costs of step-down care in their setting; their patients were predominantly reconstructed with hip replacement endoprostheses. Ashford et al. [23] reviewed the financial implications of using proximal femoral replacements for metastatic bone disease and reported that, in their setting, endoprosthetic replacements were effective treatment but poorly reimbursed under their funding arrangements. In Germany, public hospitals get reimbursed for their treatment of patients according the diagnosis-related group system. These diagnosis-related groups are assigned based on the International Classification of Diseases diagnoses, procedures, and several co-factors. The reimbursement for modular tumor endoprostheses does not differentiate between primary bone sarcomas and metastases. There is currently no homogeneous reliable data on overall economic effectiveness of the usage of modular endoprostheses in metastatic patients in Germany, but inferences can be drawn based the advancements in multimodal treatment of cancer patients and economic models used in studies of this nature in other healthcare models [22, 24]. Thus, cost remains a concern and funding arrangements differ from country to country but it seems intuitive that the cost-benefit ratio is in favor of tumor resection and replacement with a modular tumor endoprosthesis in patients with solitary large, periarticular metastatic lesions with primary cancers of a favorable histological subtype and amenable to cure.

**Figure 2:**
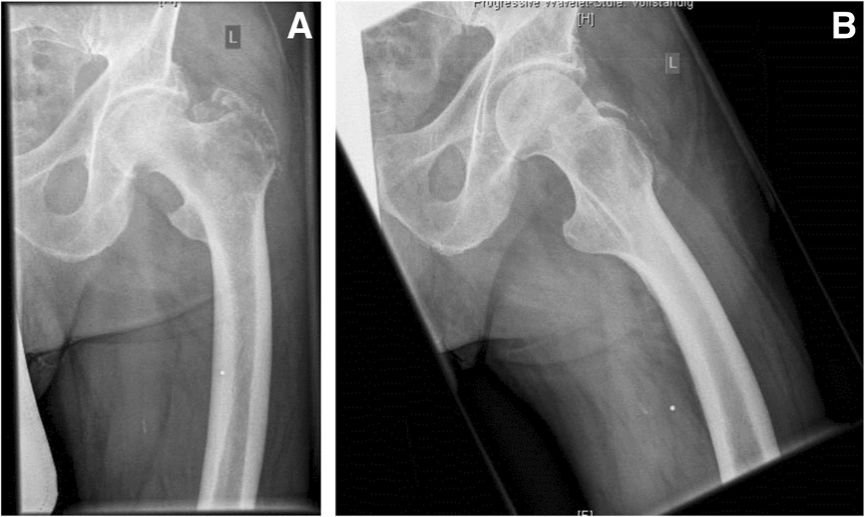
**(A) and (B) Radiographs showing a representative case of a proximal femur metastasis: (A) anterior-posterior and (B) axial radiograph of the proximal femur of a 76-year-old male patient with a renal cell carcinoma metastasis.** The metastasis lead to a pathological fracture of the proximal femur. An embolization has been performed pre-operatively to reduce intraoperative bleeding (coil visible in pre-operative radiographs).

**Figure 3:**
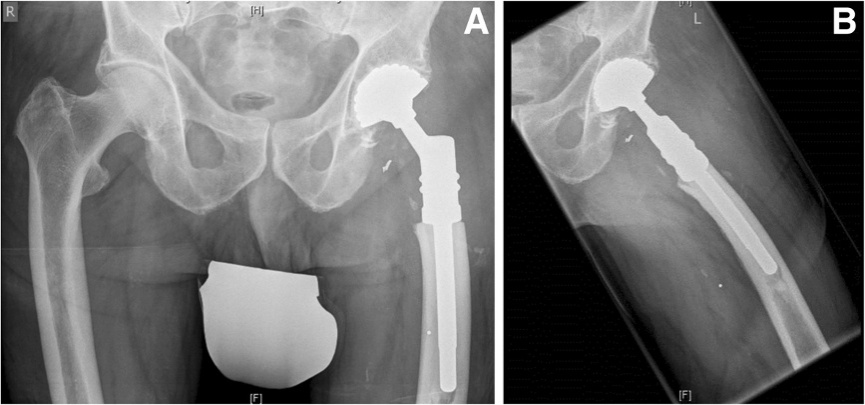
**(A) and (B): Radiographs showing the typical post-operative findings after reconstruction with a modular tumor endoprosthesis: (A) anterior-posterior and (B) axial radiograph of the proximal femur of a 76-year-old male patient (see Figure** 2 **) after resection of the fractured proximal femur.** Modular proximal femur construct with cemented stem and with cemented Avantage cup (tripolar).

To put our data into perspective, the patients who received modular tumor endoprostheses for reconstruction of metastatic disease represent a minority of the overall number of patients who presented with bone metastases in our institution. In general, these were patients with larger, solitary metastatic deposits. An example of such a case is shown in Figures 2 and 3. In terms of tumor type, renal cell and breast cancer were the predominant tumor types for whom modular endoprostheses were used. Most other patients who presented with metastases to our unit still received an osteosysthesis construct. We refrain from presenting a selection criterion for endoprostheses versus osteosynthesis as these decisions are often individualized and depicting this situation in pure black and white categories may be an oversimplification and potentially convey the wrong message to junior musculoskeletal oncologists climbing the learning curve. For simplicity, resection of long bone skeletal metastases and replacement with modular endoprostheses is preferred for large destructive solitary metastases with primary cancer type/site that is amenable to cure and/or has been resected with a curative intent. This is in keeping with data presented by Ratasvuori et al. [25], who showed that significant factors in prognosis of *en bloc* resection of kidney, breast, prostate, and lung carcinomas include the presence of organ metastases, overall health status, and disease load.

## Conclusions

Our data collectively suggest that modular tumor endoprosthetic reconstruction is a suitable option for surgical palliation of long-bone skeletal metastases in appropriately selected patients presenting with large tumors and consequent intralesional resections, recurrences, renal cell carcinoma, and solitary metastases. The ability of conventional osteosynthetic constructs to outlive the patient with metastatic cancer should be reevaluated in today’s context of increased survival as a result of multimodal cancer treatment coupled with advances in musculoskeletal oncology as a subspecialty.

## Consent

All patients signed an informed consent form at hospital admission allowing the use of anonymized data and images for research purposes.
